# Impact of Intrinsic Resistance Mechanisms on Potency of QPX7728, a New Ultrabroad-Spectrum Beta-Lactamase Inhibitor of Serine and Metallo-Beta-Lactamases in *Enterobacteriaceae*, Pseudomonas aeruginosa, and Acinetobacter baumannii

**DOI:** 10.1128/AAC.00552-20

**Published:** 2020-05-21

**Authors:** Olga Lomovskaya, Kirk Nelson, Debora Rubio-Aparicio, Ruslan Tsivkovski, Dongxu Sun, Michael N. Dudley

**Affiliations:** a Qpex Biopharma, Inc., San Diego, California, USA

**Keywords:** QPX7728, serine beta-lactamases, metallo-beta-lactamases, efflux, porins

## Abstract

QPX7728 is an ultrabroad-spectrum boronic acid beta-lactamase inhibitor that demonstrates inhibition of key serine and metallo-beta-lactamases at a nanomolar concentration range in biochemical assays with purified enzymes. The broad-spectrum inhibitory activity of QPX7728 observed in biochemical experiments translates into enhancement of the potency of many beta-lactams against strains of target pathogens producing beta-lactamases. The impacts of bacterial efflux and permeability on inhibitory potency were determined using isogenic panels of KPC-3-producing isogenic strains of Klebsiella pneumoniae and Pseudomonas aeruginosa and OXA-23-producing strains of Acinetobacter baumannii with various combinations of efflux and porin mutations. QPX7728 was minimally affected by multidrug resistance efflux pumps either in *Enterobacteriaceae* or in nonfermenters, such as P. aeruginosa or A. baumannii. Against P. aeruginosa, the potency of QPX7728 was further enhanced when the outer membrane was permeabilized. The potency of QPX7728 against P. aeruginosa was not affected by inactivation of the carbapenem porin OprD. While changes in OmpK36 (but not OmpK35) reduced the potency of QPX7728 (8- to 16-fold), QPX7728 (4 μg/ml) nevertheless completely reversed the KPC-mediated meropenem resistance in strains with porin mutations, consistent with the lesser effect of these mutations on the potency of QPX7728 compared to that of other agents. The ultrabroad-spectrum beta-lactamase inhibition profile, combined with enhancement of the activity of multiple beta-lactam antibiotics with various sensitivities to the intrinsic resistance mechanisms of efflux and permeability, indicates that QPX7728 is a useful inhibitor for use with multiple beta-lactam antibiotics.

## Abstract (precis)

QPX7728 is an ultrabroad-spectrum boronic acid beta-lactamase inhibitor that demonstrates inhibition of key serine and metallo-beta-lactamases at a nanomolar concentration range in biochemical assays with purified enzymes. The broad-spectrum inhibitory activity of QPX7728 observed in biochemical experiments translates into enhancement of the potency of many beta-lactams against strains of target pathogens producing beta-lactamases. The impacts of bacterial efflux and permeability on inhibitory potency were determined using isogenic panels of KPC-3-producing isogenic strains of Klebsiella pneumoniae and Pseudomonas aeruginosa and OXA-23-producing strains of Acinetobacter baumannii with various combinations of efflux and porin mutations.

## INTRODUCTION

**FIG 1:**
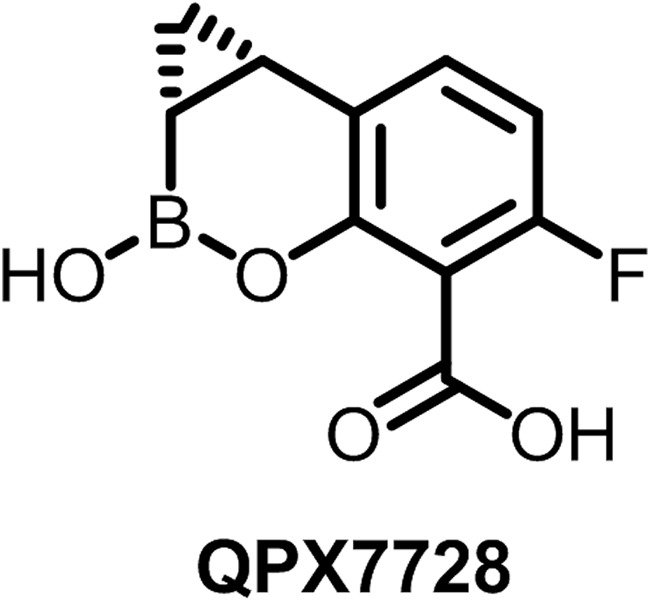
QPX7728 structure.

QPX7728 (Fig. 1) is new cyclic boronic acid beta-lactamase inhibitor (BLI) (1) that inhibits key serine beta-lactamases and metallo-beta-lactamases (MBLs) at a nanomolar concentration range in biochemical assays with purified enzymes (2). QPX7728 inhibits class A extended-spectrum beta-lactamases and carbapenemases, such as KPC, as well as the class C beta-lactamase P99 with a potency that is comparable to or higher than that of the recently FDA-approved BLIs avibactam, relebactam, and vaborbactam. Unlike those other BLIs, QPX7728 is also a potent inhibitor of class D carbapenemases, such as OXA-48 from *Enterobacteriaceae* and OXA enzymes from Acinetobacter baumannii, as well as MBLs, such as NDM-1, VIM-1, and IMP-1.

QPX7728 differs from investigational beta-lactamase inhibitors in clinical development, such as the avibactam analog durlobactam and the bicyclic boronate taniborbactam (3). The durlobactam spectrum includes OXA enzymes from Acinetobacter, and its potency for inhibition of these enzymes appears to be similar to that of QPX7728 (based on *K_d_* [dissociation constant] values), but it does not inhibit class B metallo-beta-lactamases (37, 38). Taniborbactam and QPX7728 inhibit NDM and VIM metallo-beta-lactamases with a similar potency, but taniborbactam lacks inhibitory activity against OXA carbapenemases from Acinetobacter (6, 7).

The broad-spectrum inhibitory activity of QPX7728 observed in biochemical experiments translates into enhancement of the activity of many beta-lactams against extended-spectrum-beta-lactamase- and carbapenemase (serine beta-lactamase and metallo-beta-lactamase)-producing strains of *Enterobacteriaceae*, carbapenemase (OXA)-producing strains of Acinetobacter baumannii, and multidrug-resistant (MDR) strains of Pseudomonas aeruginosa (1). This inhibitory activity of QPX7728 can also be demonstrated in mouse thigh and lung infection models of infections, where meropenem showed efficacy against KPC-producing strains of *Enterobacteriaceae* (Klebsiella pneumoniae and Enterobacter cloacae), OXA-23-producing strains of A. baumannii, and multidrug-resistant strains of P. aeruginosa that did not respond to meropenem alone (8).

Gram-negative bacteria possess multiple intrinsic mechanisms that modulate the activity of various antibiotics, including beta-lactams (9, 10). Reduced uptake across the outer membrane, increased efflux by multidrug resistance pumps, and a combination of these mechanisms result in decreased susceptibility to beta-lactam antibiotics that cannot be reversed with beta-lactamase inhibitors. In *Enterobacteriaceae*, this occurs due to mutations in the major porins OmpF/OmpK35 and OmpC/OmpK36 (4, 5, 11, 12) and increased efflux out of cells by multidrug resistance efflux pumps, such as AcrAB (13). P. aeruginosa in particular has multiple intrinsic resistance mechanisms that impact multiple antibiotics (14), notably, with several multidrug resistance efflux pumps (15), with MexAB-OprM having various effects on beta-lactam antibiotics. Mutations in the porin OprD (16) are specifically associated with reduced susceptibility to carbapenems. AdeABC and AdeIJK MDR efflux pumps from A. baumannii are implicated in general defense (17). Similar to beta-lactams, the potency of beta-lactamase inhibitors can also be affected by the same general resistance mechanisms (18–21).

The objective of this study was to investigate the impact of the general intrinsic resistance mechanisms of Gram-negative bacteria on the inhibitory potency of QPX7728. The impact of porin and efflux mutations was investigated in several target bacteria using various microbiological assays. The choice of an antibiotic in each assay presented here was driven by the intrinsic resistance mechanism being probed for QPX7728, where the partner antibiotic tested may be affected little or to no extent. The findings from studies of QPX7728 in these defined systems should help to provide a better understanding of its behavior against clinical isolates with their complicated combinations of various intrinsic resistance mechanisms.

## RESULTS AND DISCUSSION

### The outer membrane porin OmpK36 and, to a lesser degree, efflux modulate the whole-cell broad-spectrum inhibitory activity of QPX7728 in K. pneumoniae.

A set of isogenic KPC-producing strains of K. pneumoniae with various combinations of efflux and porin mutations was used to investigate the contribution of porins and efflux to the broad-spectrum inhibitory activity of QPX7728 in K. pneumoniae (22). Meropenem was used as a tool antibiotic. The effects of various concentrations of QPX7728 or vaborbactam on meropenem MICs were assessed in checkerboard experiments. As the parameter for comparison of broad-spectrum inhibitory potency, we used the concentration of a BLI required to reduce the meropenem MIC to the level seen for the parent strain that lacks KPC. This value, the maximal potentiation value (PV_max_), corresponds to the concentration that completely inhibits the enzyme.

The inactivation of *ompK35* alone did not increase the QPX7728 PV_max_s (compare the results for KPM2601 to those for KPM1271), while the inactivation of *ompK36* alone increased the QPX7728 PV_max_ 8- to 16-fold, from 0.125 μg/ml to 1 to 2 μg/ml (compare the results for KPM2599 and KPM2067 to those for KPM1271) (Table 1; see Table S2 in the supplemental material for the full concentration-response of the meropenem MIC to various concentrations of QPX7728 and vaborbactam). Vaborbactam PV_max_s were increased 4-fold and 64-fold by the inactivation of OmpK35 (to 1 μg/ml) and OmpK36 alone (to 16 μg/ml), respectively. Note that both BLIs have similar potencies against wild-type strain PAM1271. The inactivation of *ompK35* in the strain that already lacked OmpK36 (KPM2631 and KPM2067, respectively) did not increase the PV_max_ for QPX7728 (it remained at 2 μg/ml), while the vaborbactam PV_max_ was increased 4-fold, to 64 μg/ml. Inactivation of both OmpK35 and OmpK36 reduced the vaborbactam potency 256-fold; only a 16-fold effect (driven by OmpK36 alone) was observed for QPX7728.

**TABLE 1:** PV_max_ of QPX7728 for enhancement of the activity of meropenem against isogenic strains of KPC-3-producing K. pneumoniae with various combinations of efflux and porin mutations^*a*^

KPC-3-producing strain	Recipient strain	Genotype/construction	Expression			Meropenem MIC (μg/ml) for KPC-3-producing strain with:			Meropenem MIC (μg/ml) for recipient	PV_max_ (μg/ml)	
			OmpK35	OmpK36	AcrAB	No drug	QPX	VAB		QPX	VAB
KPM1271	KPM1026a	Wild type	FL	FL	BL	16	0.016	0.06	0.06	0.125	0.25
KPM2601	KPM2600	Δ*ompK*35	NF	FL	BL	32	0.016	0.06	0.06	0.125	1
KPM2599	KPM2592	Δ*ompK*36	FL	NF	BL	32	0.03	0.25	0.06	1	16
KPM2067	KPM2040	*ompK*36_fs	FL	NF	BL	64	0.03	0.5	0.125	2	16
KPM2631	KPM2613	*ompK*36_fs Δ*ompK*35	NF	NF	BL	256	0.25	4	0.25	2	64
KPM2965	KPM2966	ramR* ompK36_fs Δ*ompK*35	NF	NF	Up	256	0.5	8	0.5	4	64
KPM1272	KPM1027	*ramR*_fs	Down	FL	Up	16	0.016	0.06	0.06	0.25	2
KPM2818	KPM2658	*ramR*_fs Δ*ompK*36	Down	NF	Up	256	0.5	8	0.5	4	64
	KPM1007	Δ*acrB*	FL	FL	NF	ND	ND	ND	0.06	ND	ND
KP1004	KPM1206	*ompK*35_fs	NF	FL	BL	32	0.016	0.03	0.03	0.25	2
KP1074	KPM1211	*ompK*35_fs *ompK*36_GD	NF	GD	BL	128	0.06	1	0.125	2	32

a All strains produced KPC-3 and TEM-1, the genes for which were carried by plasmid pKpQIL. Both the KPM1026a derivatives and clinical isolates also produced a chromosomal SHV enzyme, encoded by *bla*_SHV-24_ and *bla*_SHV-11_, respectively. ompK35_fs and ompK36_fs, truncated porins due to the frameshift mutation; ramR*, truncated *ramR* due to the stop codon; FL, full length; NF, nonfunctional; BL, basal level; Up, overexpressed; Down, downregulated; QPX, QPX7728; VAB, vaborbactam at 4 μg/ml; ND, not determined; GD, duplication of two amino acids, Gly134 and Asp135, located within the L3 internal loop and associated with the reduced susceptibility to carbapenems due to constriction of the channel; PV_max_, maximal potentiating value, which is the concentration of the BLI that reduces the meropenem MIC to the level seen in the parent strain that lacks KPC, which corresponds to the complete inhibition of KPC. KPC-3-producing strain was not constructed for KPM1007.

Strains of K. pneumoniae that carry a variant of OmpK36 with a duplication of two amino acids, Gly134Asp135 in the L3 loop (GD repeat), results in a functionally constricted inner channel; these variants are increasingly being reported in clinical settings (23). The PV_max_ of QPX7728 for the potentiation of the activity of meropenem against clinical isolate KP1074 (2 μg/ml; isolate KP1074 has OmpK36 with a GD repeat) was 8-fold higher than that for the potentiation of the activity of meropenem against KP1004 (0.25 μg/ml; KP1004 has wild-type OmpK36). In the case of vaborbactam, PV_max_s were increased 16-fold, from 2 μg/ml to 32 μg/ml. While these strains are not truly isogenic, the GD repeat in KP1074 is the most likely reason for this difference. Based on the PV_max_, the narrowing of the OmpK36 aperture had the same effect on the potency of QPX7728 as the complete inactivation of OmpK36 did.

Strains KPM1272 and KPM1271 were used to assess the effect of the *ramR* mutation on the potentiation of the activity of meropenem by QPX7728. Inactivation of *ramR* (KPM1272) results in both ∼3- to 4-fold *acrAB* overexpression and ∼10-fold *ompK35* downregulation (22). The QPX7728 PV_max_ was 2-fold higher in KPM1272 than in KPM1271; the PV_max_ for KPM1272 was 2-fold higher than that for KPM2601, the mutant that lacks OmpK35. In addition, a 2-fold effect of AcrAB overexpression on QPX7728 inhibitory potency was seen in the strains that lacked (or that had low levels of expression of) both OmpK35 and OmpK36 (strains KPM2631 versus KPM2818 and strains KPM2631 versus KPM2965). These data indicate that the increased efflux due to the AcrAB efflux pump has a small effect on QPX7728 potentiation of the activity of meropenem in K. pneumoniae.

In summary, the complete or partial inactivation of the major porin OmpK36 results in reduced whole-cell broad-spectrum inhibitory potency with QPX7728. OmpK36 deficiency appears to affect QPX7728 to a significantly lesser degree than it affects vaborbactam (a 16-fold reduction versus a 64-fold reduction in the PV_max_ for QPX7728 and vaborbactam, respectively). In contrast to vaborbactam, the broad-spectrum inhibitory potency of QPX7728 is not affected by mutations in OmpK35. Consequently, there is no additional loss in the inhibitory potency of QPX7728 in strains with double porin mutations, whereas the vaborbactam potency was further reduced (256-fold decrease). Increased efflux conferred a ca. 2-fold decrease in the potency (an increase in the PV_max_) of QPX7728, so that the highest concentration of QPX7728 associated with the complete inhibition of KPC activity in whole cells was observed in the strains with a combination of increased efflux and defects in OmpK36. Even though a combination of these mechanisms resulted in a 16- to 32-fold reduction of the broad-spectrum inhibitory potency of QPX7728, the PV_max_ did not exceed 4 μg/ml; in contrast, 64 μg/ml of vaborbactam was required to completely inhibit KPC in a mutant lacking both major porins in the background of increased efflux. Since porin mutations reduce the potency of recently approved beta-lactam–BLI combinations in clinical settings (18–21), the finding that QPX7728 is affected by porin mutations to a lesser degree will be advantageous for several QPX7728 combinations.

### The broad-spectrum inhibitory activity of QPX7728 is not affected by inactivation of the OprD porin in P. aeruginosa.

Inactivation of OprD in P. aeruginosa was studied to determine the effect on QPX7728 potency. The OprD porin is used by carbapenem antibiotics (but not other beta-lactams) to cross the outer membrane, and *oprD* mutations are associated with decreased sensitivity to carbapenems in P. aeruginosa (16). The mutational inactivation of *oprD* or its reduced expression is prevalent in carbapenem-resistant isolates (24–26). The impact of OprD inactivation was probed using two isogenic KPC-producing stains, PAM4135 and PAM4756, that either carried or lacked a functional OprD (both strains also lacked MexAB-OprM), respectively (Table 2). Cefepime was used as a reporter antibiotic in the checkerboard experiments with QPX7728, as its activity is not affected by OprD inactivation. The QPX7728 PV_max_ for cefepime potentiation was not affected by OprD inactivation, indicating that OprD is not important for the uptake of QPX7728. When meropenem was used as a reporter antibiotic, inactivation of OprD, as expected, increased the meropenem MIC alone or with QPX7728 at various concentrations ca. 4-fold (Table 2). The lack of an effect of OprD inactivation, a prevalent mechanism of resistance in carbapenem-resistant isolates, on QPX7728 means that if QPX7728 is combined with an antipseudomonal beta-lactam other than a carbapenem, the potency of a combination agent will not be affected.

**TABLE 2:** Effect of inactivation of carbapenem-specific porin OprD or permeabilization of outer membrane on potency of QPX7728 against P. aeruginosa^*a*^

Strain^*b*^	Genotype	Antibiotic	Antibiotic MIC (μg/ml) in the presence of the following concn of QPX7728 (μg/ml)^*c*^:								MIC (μg/ml) for vector-only control	PV_max_ (μg/ml)
			0	0.125	0.25	0.5	1	2	4	8		
PAM4135	*oprM*::Hg	Cefepime	256	64	64	64	4	0.125	0.125	≤0.06	0.125	2
PAM4756	*oprM*::Hg *oprD*^*d*^	Cefepime	256	64	64	32	2	**0.125**	0.125	0.125	0.125	2
PAM4135	*oprM*::Hg	Meropenem	64	64	64	32	4	**0.03**	0.03	0.03	0.03	2
PAM4756	*oprM*::Hg *oprD*^*d*^	Meropenem	256	ND	ND	64	16	**0.25**	0.25	0.25	0.25	2
PAM4135	*oprM*::Hg	Meropenem (2.5)^*e*^	32	0.5	0.125	**0.03**	0.03	0.03	0.03	0.015	0.03	0.5
PAM4135	*oprM*::Hg	Meropenem (5)^*e*^	8	**0.015**	0.015	0.008	0.008	0.008	0.008	0.008	0.015	≤0.125

a The impact of OprD inactivation on the broad-spectrum inhibitory potency of QPX7728 was evaluated based on the ability of QPX7728 to reduce the cefepime or meropenem MIC in isogenic KPC-producing strains that produced or lacked OprD. The effect of the outer membrane barrier on the broad-spectrum inhibitory potency of QPX7728 was evaluated based on the ability of the outer membrane-permeabilizing agent PMBN to increase the broad-spectrum inhibitory potency of QPX7728 in a meropenem potentiation assay using the KPC-2-producing strain of P. aeruginosa PAM4135, which lacks the MexAB-OprM efflux pump. The potency of QPX7728 was expressed as PV_max_, which is the maximal potentiating value, or the concentration of the BLI that reduces the antibiotic MIC to the level seen in the parent strain that lacks KPC (corresponding to the complete inhibition of KPC).

b All strains produced KPC-2, the gene for which was carried by recombinant plasmid pUCP24-KPC-2.

c Cefepime and meropenem MICs in the presence of BLIs at PV_max_ are in boldface.

d *oprD* mutant PAM4756 (*oprD*::fs_aa126) was selected from PAM4135 on 128 μg/ml of biapenem (4× MIC).

e Meropenem-QPX7728 checkerboard plates contained PMBN at a fixed concentration of 2.5 μg/ml or 5 μg/ml, with the PMBN concentration used being indicated in parentheses.

### The inhibitory activity of QPX7728 is affected by the outer membrane barrier in P. aeruginosa.

The contribution of the outer membrane barrier of P. aeruginosa to the broad-spectrum inhibitory activity of QPX7728 was studied using strains with various efflux activities and the outer membrane-permeabilizing agent polymyxin B nonapeptide (PMBN). The QPX7728 PV_max_ for the potentiation of meropenem activity was determined in the KPC-2-producing strain PAM4135, which lacks the major constitutively expressed efflux pump MexAB-OprM. MICs were determined in the presence of increasing concentrations of the outer membrane-permeabilizing agent PMBN (27) (Table 2). PMBN at 2.5 μg/ml and 5 μg/ml reduced the MIC of meropenem 2-fold and 8-fold, respectively. In the presence of PMBN at concentrations of 2.5 μg/ml and 5 μg/ml, QPX7728 PV_max_s were reduced 4-fold and 16-fold, respectively. These data show that, like many other small-molecule antibiotics, the outer membrane of P. aeruginosa does present a barrier to the entry of QPX7728. The low permeability of the outer membrane of P. aeruginosa appears to be responsible for the lower broad-spectrum inhibitory potency of QPX7728 in P. aeruginosa than in K. pneumoniae: the QPX7728 PV_max_ for KPC inhibition was 2 to 8 μg/ml in P. aeruginosa, whereas it was 0.125 to 0.25 μg/ml in K. pneumoniae. In the presence of 5 μg/ml PMBN, the QPX7728 PV_max_ for KPC inhibition in P. aeruginosa was the same as the PV_max_ for KPC inhibition in K. pneumoniae (0.125 μg/ml). Similarly, the QPX7728 PV_max_s for KPC inhibition in P. aeruginosa were also similar to those in K. pneumoniae that lack functional porins. The PV_max_s will be taken into consideration when translating the broad-spectrum inhibitory potency observed in microbiological experiments into exposures that are expected to be associated with broad-spectrum inhibitory activity *in vivo* against target pathogens. It also means that outer membrane permeabilization might be considered as a future strategy to increase the whole-cell antibiotic potentiation activity of QPX7728.

### The broad-spectrum inhibitory activity of QPX7728 is minimally affected by MDR efflux in P. aeruginosa and A. baumannii.

A set of isogenic KPC-producing strains of P. aeruginosa overexpressing or lacking major efflux pumps (28, 29) was used to assess the effect of efflux on the inhibitory activity of QPX7728 in P. aeruginosa. Biapenem was chosen as the reporter antibiotic because its activity is minimally affected by efflux (30). Vaborbactam was used as a comparator BLI. Biapenem MICs were determined in the presence of increasing concentrations of BLIs. Broad-spectrum inhibitory potency was expressed as either PV_max_ (see above) or 50% potentiation value (PV_50_) (31), where PV_50_ was defined as the concentration of a BLI achieving 50% of the antibiotic potentiation effect, which is determined by the midpoint of the difference between the partner antibiotic MIC for the beta-lactamase-producing strain and the MIC for the vector-only control strain (the MIC middle point was calculated as the geometric mean of the above-mentioned MICs) (Table 3). Overexpression of the MexAB-OprM efflux pump increased the QPX7728 PV_50_ and PV_max_ 2-fold, from 1 μg/ml to 2 μg/ml and from 4 μg/ml to 8 μg/ml, respectively (compare the results for strains PAM4126 and PAM4224). No change in QPX7728 potency was observed when this pump was inactivated due to mutations in *mexA* (PAM4365) and *oprM* (PAM4135). In contrast, vaborbactam potency was strongly affected by either increased or decreased MexAB-OprM-mediated efflux: overexpression of MexAB-OprM reduced the vaborbactam potency 8-fold (PV_50_ increased from 8 μg/ml to 64 μg/ml), while MexAB-OprM inactivation increased its potency 16-fold (PV_50_ decreased from 8 μg/ml to 0.5 μg/ml). QPX7728 potency was not affected by overexpression of the MexEF-OprN efflux pump, whereas vaborbactam potency was significantly reduced (>8-fold increase in the PV_50_ to >64 μg/ml, as seen in the comparison of strains PAM4132 and PAM4224). Neither MexXY-OprM nor MexCD-OprJ had a significant effect on the activity of either BLI.

**TABLE 3:** PV_50_ and PV_max_ of QPX7728 for enhancement of activity of biapenem against isogenic strains of KPC-2-producing P. aeruginosa overexpressing or lacking MDR RND efflux pumps

Strain^*a*^	Genotype/description	BLI	Biapenem MIC (μg/ml) in the presence of the following concn of BLIs (μg/ml)^*b*^:												Biapenem MIC (μg/ml) for vector-only control	GM biapenem MIC^*c*^ (μg/ml)	PV_50_^*d*^ (μg/ml)	PV_max_^*e*^ (μg/ml)
			0	0.06	0.13	0.25	0.5	1	2	4	8	16	32	64				
PAM4224	Wild type	QPX7728	64	64	32	32	16	*2*	1	**0.06**	0.06	0.06	0.06	0.03	0.25	4	1	4
PAM4135	*oprM*::Hg	QPX7728	64	32	32	32	16	*2*	0.5	**0.06**	0.03	NG	NG	NG	0.25	4	1	4
PAM4365	*mexA*::Tet	QPX7728	64	32	32	16	8	*2*	0.5	**0.125**	0.125	NG	NG	NG	0.25	4	1	4
PAM4126	*mexR* (MexAB-OprM)^*f*^	QPX7728	64	16	16	16	16	4	*1*	0.25	**0.125**	0.125	0.125	0.125	0.125	2.8	2	8
PAM4129	*nfxB* (MexCD-OprJ)	QPX7728	8	8	4	4	*0.5*	0.06	**0.03**	0.03	0.03	0.03	0.3	NG	0.03	0.5	0.5	2
PAM4132	*mexT* (MexEF-OprM)^*g*^	QPX7728	128	128	128	64	32	*8*	2	**0.5**	0.25	0.25	0.25	0.125	0.5	8	1	4
PAM4150	*mexZ* (MexXY-OprM)	QPX7728	64	32	32	16	8	*2*	1	**0.25**	0.25	0.25	0.25	0.125	0.25	4	1	4
PAM4224	Wild type	Vaborbactam	64	64	64	64	32	32	16	8	*4*	2	2	1	0.25	4	8	>64
PAM4135	*oprM*::Hg	Vaborbactam	64	32	32	16	*4*	2	0.5	0.5	**0.25**	0.25	0.25	0.25	0.25	4	0.5	8
PAM4365	*mexA*::Tet	Vaborbactam	64	32	32	16	*4*	2	1	**0.5**	0.25	0.25	0.25	0.25	0.25	4	0.5	4
PAM4126	*mexR* (MexAB-OprM)	Vaborbactam	64	64	64	64	64	64	64	16	16	8	4	*2*	0.125	2.8	64	>64
PAM4129	*nfxB* (MexCD-OprJ)	Vaborbactam	8	8	8	8	4	2	1	*0.5*	0.25	0.125	0.06	**0.03**	0.03	0.5	4	64
PAM4132	*mexT* (MexEF-OprM)	Vaborbactam	128	32	32	32	32	32	32	32	32	32	32	64	0.5	8	>64	>64
PAM4150	*mexZ* (MexXY-OprM)	Vaborbactam	64	64	64	32	32	32	16	8	*4*	4	2	1	0.25	4	8	>64

a All strains produced KPC-2, the gene for which was carried by recombinant plasmid pUCP24-KPC-2.

b Biapenem MICs in the presence of BLIs at PV_50_ and PV_max_ are marked in italics and boldface, respectively. NG, no growth.

c GM MIC, the geometric mean of the antibiotic MIC values for the beta-lactamase-producing and the vector-only control strain (calculated as the square root of the product of the antibiotic MIC values for the beta-lactamase-producing and the vector-only control strain).

d PV_50_, the concentration of a BLI required to achieve a 50% antibiotic potentiation effect or the concentration of a BLI required to reduce the antibiotic MIC to or below the middle point of the MIC range between the MIC for the beta-lactamase-producing strain and the MIC for the vector-only control strain. The MIC middle point is the geometric mean of the antibiotic MIC values for the beta-lactamase-producing and the vector-only control strain.

e PV_max_, maximal potentiating value, or the concentration of the BLI that reduces the antibiotic MIC to the level seen in the parent strain that lacks KPC (corresponding to the complete inhibition of KPC).

f The specific efflux pump overexpressed due to mutations in respective regulators is shown in parentheses.

g A mutation in *mexT* also results in a partial downregulation of the carbapenem-specific porin *oprD*.

The results of these experiments demonstrate that the broad-spectrum inhibitory activity of QPX7728 is minimally affected by the activity of major MDR efflux pumps from P. aeruginosa, representing a significant improvement over the earlier-generation boronate BLI vaborbactam. As this result was obtained using KPC as a reporter beta-lactamase, we wanted to test the validity of this conclusion using an additional beta-lactamase and more antibiotics. Overexpression of chromosomal AmpC is one of the more prevalent mechanisms of beta-lactam resistance in P. aeruginosa (14). Hence, we evaluated the MICs of several beta-lactam antibiotics in combination with QPX7728 against isogenic strains of P. aeruginosa overexpressing the chromosomal AmpC beta-lactamase (PDC-1) with or without concomitant overexpression of the major efflux pump MexAB-OprM. QPX7728 was tested at 4 μg/ml and 8 μg/ml, the latter of which corresponds to the PV_max_ of QPX7728 for the inhibition of KPC in the strain overexpressing MexAB-OprM.

The lowest MIC increase due to AmpC overexpression, 4-fold (from 0.5 μg/ml to 2 μg/ml), was observed for meropenem, and the highest (>64-fold; from 4 μg/ml to >256 μg/ml) was observed for piperacillin (these changes were found in strains PAM1020 and PAM2156, respectively) (Table 4). Ceftolozane MICs were not affected by MexAB-OprM-mediated efflux, and other antibiotics were affected 4- to 8-fold (see the results for PAM1020 versus those for PAM1032). Overexpression of AmpC in the strain with MexAB-OprM overexpression did not significantly increase the MIC values of meropenem; the MIC values of other antibiotics were increased 4- to >16-fold (see the results for PAM1032 versus those for PAM2005). QPX7728 (tested at either 4 μg/ml or 8 μg/ml) had no effect on antibiotic MICs against PAM1020 and PAM1032; however, when tested against strain PAM2156, which overexpressed AmpC but which had a basal level of MexAB-OprM, QPX7728 at both concentrations reduced the MIC values of all tested antibiotics except piperacillin to the level observed for PAM1020, indicating the complete inhibition of AmpC. The MIC of piperacillin tested with QPX7728 at 4 μg/ml was 4-fold higher for PAM2156 than for PAM1020 (16 μg/ml versus 4 μg/ml); increasing the QPX7728 concentration to 8 μg/ml resulted in a complete reversion of resistance, presumably due to the complete inhibition of AmpC. For strain PAM2005, overexpressing both AmpC and MexAB-OprM, the MICs of all antibiotics (except piperacillin) with QPX7728 at both concentrations were either the same as or not more than 2-fold higher than those for PAM1032, overexpressing MexAB-OprM alone. The MIC of piperacillin with QPX7728 at 4 μg/ml and 8 μg/ml was 8-fold and 2-fold higher, respectively, for PAM2005 than for PAM1032, indicating a very similar dose-response for potentiation compared to that observed for PAM2156 with a basal level of MexAB-OprM. These experiments confirmed that the inhibitory potency of QPX7728 for the reversal of resistance to multiple antipseudomonal beta-lactams was minimally affected by overexpression of the major efflux pumps in P. aeruginosa. This important feature differentiates QPX7728 from avibactam (32) and vaborbactam, which are substrates of efflux pumps and which demonstrate reduced potency in strains with increased efflux. Combining QPX7728 with a beta-lactam antibiotic not affected by efflux will ensure the potency of a combination agent not affected by efflux as well.

**TABLE 4:** MICs of various beta-lactam antibiotics alone and in combination with QPX7728 against isogenic strains of P. aeruginosa overexpressing MexAB-OprM and/or chromosomal AmpC^*a*^

Strain	MexAB-OprM	AmpC^*b*^	MIC (μg/ml)														
			Meropenem			Ceftolozane			Cefepime			Ceftazidime			Piperacillin		
			Alone	With QPX7728 at:		Alone	With QPX7728 at:		Alone	With QPX7728 at:		Alone	With QPX7728 at:		Alone	With QPX7728 at:	
				4 μg/ml	8 μg/ml		4 μg/ml	8 μg/ml		4 μg/ml	8 μg/ml		4 μg/ml	8 μg/ml		4 μg/ml	8 μg/ml
PAM1020	BL	1	0.5	0.5	0.5	0.5	0.5	0.5	0.5	0.5	0.5	1	1	1	4	4	4
PAM2156	BL	489	2	0.5	0.5	4	0.5	0.5	8	0.5	0.5	32	1	1	>256	16	4
PAM1032	Up	0.8	2	2	2	0.5	0.5	0.5	4	4	4	4	4	4	16	16	16
PAM2005	Up	769	4	2	2	4	1	0.5	16	4	4	64	8	8	>256	128	32

a QPX7728 was used at a fixed concentration of 8 μg/ml. All strains are derivatives of PAM1020 (PAO1). BL, basal level; Up, overexpressed. Overexpression of the MexAB-OprM efflux pump in PAM1032 and PAM2005 is due to the L75R amino acid substitution in the MexR protein. PAM2005 was selected from PAM1032 on piperacillin at 64 μg/ml. It has AmpC overproduced due to the D135H amino acid substitution in the AmpR protein. PAM2156 was obtained by transducing a piperacillin resistance marker from PAM1032 into PAM1020 using phage F116, as described in Materials and Methods. The QPX7728 MIC against all strains was >128 μg/ml.

b The numbers correspond to the level of expression of the chromosomal *ampC* relative to that in PAM1020.

QPX7728 is a potent inhibitor of class D carbapenemases from A. baumannii. Non-beta-lactamase-mediated resistance mechanisms in Acinetobacter were assessed to determine the potency of various beta-lactam–QPX7728 combinations. The impact of MDR efflux on the antibiotic potentiation activity of QPX7728 in A. baumannii (17) was evaluated using a panel of isogenic OXA-23-producing strains with wild-type major efflux operons *adeIJK* and *adeABC* (strain ACM1565) or increased expression of *adeIJK* (strain ACM1566) and *adeABC* (strain ACM1567). The QPX7728 PV_50_s and PV_max_s were determined using biapenem (which is not a substrate of efflux pumps) or meropenem potentiation experiments. QPX7728 PV_max_s for OXA-23 inhibition in A. baumannii were in the range of 2 to 4 μg/ml. When meropenem, a substrate of AdeIJK, was used as a reporter antibiotic, the overexpression of AdeIJK increased the meropenem MIC with QPX7728 at various concentrations ca. 2- to 32-fold. No changes in QPX7728 PV_max_s in either biapenem or meropenem checkerboard experiments were detected in strains overexpressing the major efflux operons, indicating that efflux has a minimal effect on the inhibitory activity of QPX7728 in A. baumannii (Table 5).

**TABLE 5:** Effects of various concentrations of QPX7728 on biapenem MICs in isogenic OXA-23-producing strains of Acinetobacter baumannii overexpressing efflux pumps

Strain^*a*^	Description	Antibiotic	Antibiotic MIC (μg/ml) in the presence of the following QPX7728 concn (mg/ml)^*b*^:							MIC (μg/ml) for recipient	PV_max_^*c*^ (μg/ml)
			0	0.25	0.5	1	2	4	8		
ACM1565	Wild type	Biapenem	32	32	16	2	1	**0.06**	0.016	0.125	4
ACM1566	AdeIJK overexpressed	Biapenem	16	16	4	2	0.5	**0.06**	0.06	0.125	4
ACM1567	AdeABC overexpressed	Biapenem	32	32	16	8	0.5	**0.06**	0.016	0.125	4
ACM1565	Wild type	Meropenem	32	32	16	4	4	**0.25**	0.03	0.5	4
ACM1566	AdeIJK overexpressed	Meropenem	32	32	32	4	**2**	2	1	2	2
ACM1567	AdeABC overexpressed	Meropenem	64	64	64	32	1	**0.25**	0.016	0.5	4

a All strains produced OXA-23, the gene for which was carried on a plasmid. Strains ACM1565, ACM1566, and ACM1567 were constructed by conjugating an OXA-producing plasmid from clinical strain AB1387 into rifampin-resistant derivatives of AB1007 (ACM1139, which is a wild-type strain), ACM1027 (ACM1494, which is an *adeN* mutant overexpressing AdeIJK), and ACM1030 (ACM1495, which is an *adeS* mutant overexpressing AdeABC), respectively.

b Biapenem and meropenem MICs in the presence of QPX7728 at PV_max_ are in boldface.

c PV_max_, maximal potentiating value, which is the concentration of QPX7728 that reduces the biapenem or meropenem MIC to the level seen in the parent strain that lacks OXA-23 (corresponding to the complete inhibition of OXA-23).

### Summary.

QPX7728 is a new boronate BLI with potent inhibitory activity against both serine and metallo-beta-lactamases. The broad-spectrum inhibitory activity of QPX7728 previously observed in cell-free biochemical experiments using purified enzymes translates into enhancement of the activity of many beta-lactams against strains of target pathogens producing beta-lactamases (2, 33).

The potent inhibitory activity of QPX7728 in whole cells is driven in part by a lack of efflux by major transporters from Gram-negative bacteria at concentrations that are relevant for beta-lactamase inhibition. A lack of efflux of QPX7728 is particularly important for inhibitory activity in P. aeruginosa and represents a significant improvement over the earlier boronate BLI vaborbactam. Mutations in outer membrane porin proteins of *Enterobacteriaceae* are associated with the reduced potency of many antibiotics and beta-lactamase inhibitors. The potency of QPX7728 in *Enterobacteriaceae* is affected by the inactivation of the major general porins OmpK35/OmpF and OmpK36/OmpC much less than the boronate inhibitor vaborbactam is.

The potent, ultrabroad-spectrum inhibitory activity of QPX7728 shown with multiple beta-lactam antibiotics with various sensitivities to beta-lactamases as well as intrinsic resistance mechanisms makes it an ideal candidate for multiple product development strategies. Conventional approaches for product configurations include the development of a fixed-combination beta-lactam–beta-lactamase inhibitor for which there is a well-established regulatory path. An important limitation of this strategy is identifying a partner beta-lactam that, in combination with the BLI, has the best overall activity against most but perhaps not all target pathogens with different mixtures of resistance mechanisms. Another approach would be the development of QPX7728 as a stand-alone drug product that could be coadministered with different existing beta-lactam antibiotics, depending on the mechanisms present in the specific pathogen. This approach has several clinical and regulatory implications but could be an important step toward individualized treatment of infections caused by drug-resistant pathogens by taking into account local epidemiology, patient factors, and antibiotic stewardship. The multiple benefits of this strategy should encourage the establishment of a defined path for future regulatory approval.

## MATERIALS AND METHODS

### Panels of engineered bacterial strains containing various combinations of porin and efflux mutations.

The efflux/porin isogenic panels of K. pneumoniae, P. aeruginosa, and A. baumannii strains were constructed to evaluate the impact of various molecular determinants on the whole-cell antibiotic potentiation activity of QPX7728.

The construction of a panel of isogenic KPC-3-producing strains (in which KPC-3 was carried on a naturally occurring plasmid, pKpQIL) of K. pneumoniae with various combinations of porin (*ompK35* and *ompK36*) and efflux (*acrAB-tolC*) mutations was described earlier (22). The panel of isogenic KPC-2-producing strains of P. aeruginosa overexpressing or lacking MDR RND efflux pumps MexAB-OprM, MexCD-OprJ, MexEF-OprN, and MexXY-OprM and producing or lacking the carbapenem porin OprD was constructed by transforming plasmid pUCP24-KPC-2 into various mutants. The panel of isogenic OXA-23-producing strains of A. baumannii overexpressing MDR RND efflux pumps AdeABC and AdeIJK was constructed by conjugating the natural plasmid that carries OXA-23 from clinical isolate AB1177 into various efflux mutants.

A detailed description of all strains used in this study is provided in Table S1 in the supplemental material.

### Conjugation in Acinetobacter baumannii.

First, rifampin-resistant mutants of recipient strains were isolated on Luria-Bertani (LB) agar containing rifampin at 40 μg/ml. Next, both donor and recipient strains were grown overnight in LB medium at 37°C with aeration. On the next day, the donor and recipient cultures (100 μl each) were mixed and pelleted by centrifugation for 1 min at room temperature. The cells were resuspended in 40 μl of LB medium and spotted onto an LB agar plate without antibiotics. Recipient-only and donor-only cultures were similarly spotted onto LB plates as negative controls. The plates were incubated at 37°C for 4 to 5 h, and cells were collected and resuspended in 0.5 ml of LB medium to an optical density at 600 nm (OD_600_) of 0.1 to 0.5. Then, 0.05 to 0.1 ml of cell suspension was plated on a plate containing rifampin and meropenem at 40 μg/ml and 2 μg/ml, respectively. Transconjugants were verified by PCR and DNA sequencing.

### Transduction in P. aeruginosa using phage F116.

Lysates of the donor strains were prepared using the soft agar method, with modifications (34). Briefly, 0.1 ml of a culture grown overnight in LB broth was mixed with 1 ml of the F116 phage suspension with a titer of 5 × 10^4^ PFU per ml in LB broth. The mixtures were incubated at 37°C for 15 min, and 2 ml of the melted 0.9% LB agar kept at 45°C was added to each mixture, the components were mixed, and the mixture was immediately poured onto a freshly prepared regular LB agar plate. The plates were incubated right side up overnight at 30°C. The top soft agar layer was scraped off, after adding 5 ml of LB broth, and poured into a centrifuge tube. Two drops of chloroform was added to each tube. The tubes were vortexed vigorously for 1 min, kept at 4°C for 1 h, and centrifuged at 5,000 rpm for 5 min at 4°C. The supernatant was sterilized by filtration. For transduction, recipient cells were grown in LB medium to an OD_600_ of 1, and 0.3 ml of the culture was mixed with 15 μl of a phage lysate prepared from a donor strain. After incubation at 37°C for 20 min, the cells were pelleted by centrifugation for 1 min and washed once with 1 ml of TNM buffer (0.01 M Tris-HCl, pH 7.4, 0.15 M NaCl, 0.01 M MgSO_4_). The cells were resuspended in 0.1 ml of TNM buffer and spread onto an LB agar plate. After 2 h of incubation at 37°C, 0.4 ml of LB broth was added to the plate and the cells were collected and directly transferred to an LB agar plate containing the selecting antibiotic. Transductants were verified by PCR and DNA sequencing.

### *ampC* gene expression.

A single colony from an overnight plate was inoculated into cation-adjusted Mueller-Hinton broth (CA-MHB) and grown at 37°C with aeration to an OD_600_ of ∼0.7. The cell culture (1.5 ml) was pelleted by centrifugation, and total RNA was isolated using an Ambion RiboPure-Bacteria RNA isolation kit (Thermo Fisher, San Diego, CA). The residual DNA in the RNA samples was removed by treatment with DNase I, according to the manufacturer’s instructions. Reverse transcription (RT) was performed using a TaqMan reverse transcriptase reagent kit (Thermo Fisher, San Diego, CA) and a mixture of reverse primers for the *ampC* gene (primer PA-ampC-R [5′-TGAAGGTCTTGCTCACCGAG-3′]) and the *polA* gene (primer PA-polA-R [5′-ATCTGGTCGAAGGTCAGTTG-3′]), each at a final concentration 0.5 μM. The RT reaction mixture was diluted 10-fold and used as a template in a quantitative PCR (qPCR) on an ABI Prism 7000 sequence detection system (Applied Biosystems) using SYBR Select master mix (Thermo Fisher). The total volume of each reaction mixture was 20 μl, including 9 μl of diluted RT reaction mixture, 10 μl of SYBR Select master mix (2×), and 1 μl of a qPCR primer pair mix (primers PA-ampC-F [5′-GAAAGGAGAACCGCATTAC-3′] and PA-ampC-R [5′-TGAAGGTCTTGCTCACCGAG-3′] and primers PA-polA-F [5′-ATCCGAAGAAGCTCAAGGTC-3′] and PA-polA-R [5′-ATCTGGTCGAAGGTCAGTTG-3′]) at a final concentration of 0.5 μM. The qPCR was run in duplicate under the following thermal cycling conditions: 50°C for 2 min and 95°C for 5 min, followed by 40 cycles of 95°C for 15 s, 55°C for 15 s, and 70°C for 45 s. The housekeeping gene *polA*, encoding DNA polymerase I, was used as an internal control. The threshold cycle (*C_T_*) value of the *ampC* gene was normalized to that of the *polA* gene of the same strain. To calculate the *ampC* gene transcription level in a test strain relative to that in wild-type strain PAM1020, the normalized *C_T_* value of PAM1020 was subtracted from that of the test strain, and the difference (Δ*C_T_*) was used as a logarithmic power (base 2) to calculate the relative level of mRNA.

### Antimicrobial susceptibility testing.

Bacterial isolates were subjected to broth microdilution susceptibility testing, performed according to Clinical and Laboratory Standards Institute (CLSI) methods (35), using panels prepared in-house. A checkerboard assay conforming to the Moody procedures in the *Clinical Microbiology Procedures Handbook* (36) was used to evaluate the effect of various concentrations of QPX7728 or vaborbactam on the MICs of various antibiotics. PV_50_ and the maximal potentiation value (PV_max_) were used to define the potencies of the beta-lactamase inhibitors (31). PV_50_ was defined as the minimal concentration of a BLI required to achieve 50% of the antibiotic potentiation effect or a concentration of a BLI to reduce the antibiotic MIC to the middle point of the MIC range between the MIC for the beta-lactamase-producing strain and the MIC for the corresponding the beta-lactamase-lacking strain. The MIC middle point is the geometric mean of the antibiotic MIC values for the beta-lactamase-producing strain and the beta-lactamase-lacking strain and is calculated as the square root of the product of the antibiotic MIC values for the beta-lactamase-producing and the beta-lactamase-lacking strain.

PV_max_ was defined as the maximal potentiating value, which was the concentration of the BLI required to reduce the antibiotic MIC to the level seen in the parent strain that lacks beta-lactamase (KPC) (corresponding to the complete inhibition of KPC).

Meropenem was purchased from Sandoz, and all other antibiotics were from Sigma-Aldrich. QPC7728 and vaborbactam were synthesized at Qpex Biopharma, Inc., San Diego, CA.

## Supplementary Material

Supplemental file 1
