# Supplementary material for: Impact of Intrinsic Resistance Mechanisms on Potency of QPX7728, a New Ultrabroad-Spectrum Beta-Lactamase Inhibitor of Serine and Metallo-Beta-Lactamases in Enterobacteriaceae, Pseudomonas aeruginosa, and Acinetobacter baumannii
Source: Antimicrob Agents Chemother. 2020 May 21;64(6):e00552-20. doi: 10.1128/AAC.00552-20 (PMC7269478; doi:10.1128/AAC.00552-20)
Supplement: Supplemental file 1 [file AAC.00552-20-s0001.pdf]

Supplementary Tables

Table S1. Strains used in this study

Strains	Acquired Beta-lactamase	Parent strain/ Recipient	Relevant genotype	Reference
Klebsiella pneumoniae				
Isogenic panel[1]				
KPM1026a	none	ATCC 43816	Wild type	(1)
KPM1271	**KPC-3**[2]**,** TEM-1	KPM1026a	Wild type	(1)
KPM1027	none	KPM1004	*ramR_fs*[3]_aa46	(1)
KPM1272	**KPC-3,** TEM-1	KPM1027	*ramR_fs*_aa46	(1)
KPM2067	**KPC-3**, TEM-1	KPM1271	*ompK36_fs_aa54*	(1)
KPM2040	none	KPM2067	*ompK36_fs_aa54*	(1)
KPM2600	none	KPM1026a	*ΔompK35*	(1)
KPM2601	**KPC-3**, TEM-1	KPM2600	*ΔompK35*	(1)
KPM2613	none	KPM2040	*ompK36_ fs_aa54 ΔompK35*	(1)
KPM2631	**KPC-3**, TEM-1	KPM2613	*ompK36_ fs_aa54 ΔompK35*	(1)
KPM2966	none	KPM2613	*ramR*_TAA[4]_aa164 *ompK36_ fs_aa54 ΔompK35*	(1)
KPM2965	**KPC-3**, TEM-1	KPM2631	*ramR_TAA*_aa164 *ompK36_ fs_aa54 ΔompK35*	(1)
KPM2592	none	KPM1026a	*ΔompK36*	(1)
KPM2599	**KPC-3**, TEM-1	KPM2592	*ΔompK36*	(1)
KPM2658	none	KPM1027	*ramR_fs*_aa46 *ΔompK36*	(1)
KPM2818	**KPC-3**, TEM-1	KPM2658	*ramR_fs*_aa46 *ΔompK36*	(1)
Clinical strains				
KP1074	**KPC-3**, TEM-1	Clinical isolate	*ompK35*_fs_aa42 *ompK36*_GD[5]	(1)
KP1004	**KPC-3**, TEM-1	Clinical isolate	*ompK35*_fs_aa42	(1)
Pseudomonas aeruginosa isogenic efflux pane				
PAM1020	none	PA01	*Wild type*	(2)
PAM1154	none	PAM1020	*oprM::Hg*	(2)
PAM1106	none	PAM1020	*mexA::Tet*	(2)
PAM1032	none	PAM1020	*mexR::L75R*	(2)
PAM1033	none	PAM1020	*nfxB ::T39I*	(2)
PAM1034	none	PAM1020	*mexT::R242P*	(2)
PAM1323	none	PAM1020	*mexZ::Δ(aa#26-39)*	(2)
PAM2005[7]	none	PAM1032	*mexR:: L75R ampR::L75R*	This study
PAM2156[8]	none	PAM1020 X PAM2005	*ampR::L75R*	This study
PAM4224	KPC-2[6]	PAM1020	*oprM::Hg*	This study
PAM4135	KPC-2	PAM1154	*mexA::Tet*	This study
PAM4365	KPC-2	PAM1106	*mexR:: L75R*	This study
PAM4126	KPC-2	PAM1032	*nfxB :: T39I*	This study
PAM4129	KPC-2	PAM1033	*mexT::R242P*	This study
PAM4132	KPC-2	PAM1034	*mexZ::Δ(aa#26-39)*	This study

PAM4150	KPC-2	PAM1323	*oprM::Hg*	This study
Acinetobacter baumannii panel[9]				
AB1187	OXA-23	Clinical isolate	*Wild-type*	Qpex collection
AB1007	none	Clinical isolate	*Wild-type*	Qpex collection
ACM1027	none	AB1007	*adeN_fs_aa156*	This study
ACM1030	none	AB1007	*adeS::P154T*	This study
ACM1139	none	AB1007 (AB1007 rifR)	*Wild-type*	This study
ACM1494	none	ACM1027 (ACM1027 riR)	*adeN_fs_aa156*	This study
ACM1495	none	ACM1030 (ACM1030 rifR)	*adeS::P154T*	This study
ACM1565	OXA-23	AB1187 X ACM1139	*Wild-type*	This study
ACM1566	OXA-23	AB1187 X ACM1494	*adeN_fs_aa156*	This study
ACM1567	OXA-23	AB1187 X ACM1495	*adeS::P154T*	This study
ACM1010	none	Clinical isolate	*adeR*	(3)
ACM1013	none	ACM1010	*adeR ΔadeIJK*	(3)
ACM1014	none	ACM1010	*ΔadeABC*	(3)
ACM1015	none	ACM1010	*ΔadeIJK ΔadeABC*	(3)

[1]All isogenic strains of *K. pneumoniae* are derivatives of KPM1026a which is a streptomycin resistant mutant of ATCC of ATCC43816.

[2]All KPC-3-producing strains were constructed by conjugating the plasmid pKpQIL from the clinical isolate of *K. pneumoniae* KP1074 (ATCC BAA-2814) into various isogenic derivatives of KPM1026a. The *bla*TEM-1 gene is located on pKpQIL plasmid together with $bla_{KPC\text{-}3}$

[3] fs_aa#XX, frame-shift at amino acid no. XX, that results in non-functional protein

[4] TAA_aa#XX, stop codon at amino acid no. XX, that results in non-functional protein

[5] Duplication of two amino acids, Gly134 and Asp135, located within the L3 internal loop and associated with the reduced susceptibility to carbapenems due to constriction of the channel.

[6]All KPC-2-producing strains of *P. aeruginosa* were constructed by transforming of pUCP-24::KPC-2 into various isogenic derivatives of PAM1020.

[7] PAM2005 was selected from PAM1032 on piperacillin at 64 µg/ml. It has AmpC overproduced due to the D135H amino acid substitution in the AmpR protein.

[8] PAM2156 was obtained by transducing Pip-R marker from PAM1032 into PAM1020 using phage F116 as described in Methods section.

[9] Isogenic strains of *A. baumannii* are derivatives of AB1007, antibiotic susceptible clinical isolates. ACM1027 and ACM1030 are *adeN* and *adeS* mutants, respectively, that were selected from AB1007 on tigecycline at 0.5 µg/ml. ACM1139, ACM1494 and ACM1495 are rifampicin resistant mutants of AB1007, ACM1027 and ACM1030, respectively (selected on rifampicin at 100 µg/ml). ACM1565, ACM1566 and ACM1566 were constructed by conjugating OXA-23 containing plasmid from AC1187 into ACM1139, ACM1494 and ACM1495, respectively.

Table S2. Effect of varying concentrations of QPX7728 or vaborbactam on meropenem MICs in isogenic KPC-3 producing strains of *K. pneumoniae with* efflux and porin mutations

KPC-3 strain[1]	Genotype	BLI	Meropenem MIC (µg/ml) in the presence of varied concentrations of BLIs (µg/ml)													PV_{max}
			0	*0.06*	*0.125*	*0.25*	*0.5*	*1*	*2*	*4*	*8*	*16*	*32*	*64*	*128*	
KPM1271	wild type	VAB	**16**	0.25	0.25	0.06	0.06	0.06	0.06	0.06	0.06	0.06	0.06	0.06	0.06	**0.25**
		QPX	**16**	0.125	0.03	0.03	0.03	0.03	0.03	0.016	ND	ND	ND	ND	ND	**0.125**
KPM2601	*ΔompK35*	VAB	**16**	2	1	0.5	0.125	0.06	0.06	0.06	0.06	0.06	0.06	0.06	0.06	**1**
		QPX	**32**	0.5	0.06	0.03	0.03	0.03	0.03	0.016	ND	ND	ND	ND	ND	**0.125**
KPM2599	*ΔompK36*	VAB	**32**	16	16	8	8	4	0.5	0.25	0.125	0.06	0.06	0.06	0.06	**16**
		QPX	**32**	32	8	4	0.25	0.06	0.03	0.03	ND	ND	ND	ND	ND	**1**
KPM2067	*ompK36FS54*	VAB	**32**	32	16	16	16	8	1	0.5	0.125	0.06	0.06	0.06	0.06	**16**
		QPX	**64**	64	64	8	4	0.25	0.03	0.03	ND	ND	ND	ND	ND	**2**
KPM2631	*Δomp35 ompK36FS54*	VAB	**256**	256	256	128	128	64	16	4	1	0.5	0.5	0.25	0.125	**64**
		QPX	**256**	256	256	128	256	128	1	0.25	0.125	ND	ND	ND	ND	**4**
KPM2965	*ramR ΔompK35 ompK36FS54*	VAB	**256**	256	256	128	128	64	32	8	2	1	0.5	0.5	0.25	**64**
		QPX	**256**	256	256	256	128	64	4	0.5	0.5	ND	ND	ND	ND	**4**
KPM4019	*ΔacrB*	QPX	**32**	0.06	0.06	0.03	0.03	0.03	0.03	0.016	0.004	NG	ND	ND	ND	**0.06**
KPM1272	*ramR*	VAB	**16**	8	2	2	0.5	0.25	0.06	0.06	0.06	0.06	0.06	0.06	0.06	**2**
		QPX	**16**	4	0.125	0.06	0.03	0.03	0.016	0.016	ND	ND	ND	ND	ND	**0.25**
KPM2818	*ramR ΔompK36*	VAB	**256**	256	256	128	64	32	16	8	2	1	1	0.5	0.5	**64**
		QPX	**256**	256	256	128	128	64	1	0.5	0.5	ND	ND	ND	ND	**4**
KP1004	*ompK35FS42*	VAB	**32**	4	4	2	0.5	0.125	0.03	0.03	0.03	0.03	0.03	0.03	0.03	**2**
		QPX	**32**	8	1	0.03	0.016	0.016	0.016	0.016	ND	ND	ND	ND	ND	**0.25**
KP1074	*ompK35FS42 OmpK36GD*	VAB	**128**	128	128	64	64	64	8	1	0.5	0.25	0.125	0.125	0.125	**32**
		QPX	**128**	128	128	128	16	0.5	0.06	0.06	ND	ND	ND	ND	ND	**2**

[1] All strains produce KPC-3 and TEM-1 encoded in plasmid pKpQIL. Both KPM1026a derivatives and clinical isolates also produce chromosomal SHV enzyme, encoded by *bla*$_{SHV-24}$. and *bla*$_{SHV-11}$, respectively.

[2] PV_{max}, minimal potentiating concentration of the BLI to reduce meropenem MIC to the level seen in the parent strain that lacks KPC, which corresponding to complete inhibition of KPC.

[3] FS, Frame-shift in OmpK35 or OmpK36 sequence at the respective amino acid.
Meropenem MIC in the presence of BLIs at PV_{max} are marked with yellow or green color for vaborbactam and QPX7728, respectively

References

1. Lomovskaya O, Sun D, Rubio-Aparicio D, Nelson K, Tsivkovski R, Griffith DC, Dudley MN. 2017. Vaborbactam: Spectrum of Beta-Lactamase Inhibition and Impact of Resistance Mechanisms on Activity in Enterobacteriaceae. Antimicrob Agents Chemother 61:e01443-17.
2. Lomovskaya O, Lee A, Hoshino K, Ishida H, Mistry A, Warren MS, Boyer E, Chamberland S, Lee VJ. 1999. Use of a genetic approach to evaluate the consequences of inhibition of efflux pumps in Pseudomonas aeruginosa. Antimicrob Agents Chemother 43:1340-6.
3. Coyne S, Courvalin P, Perichon B. 2011. Efflux-mediated antibiotic resistance in Acinetobacter spp. Antimicrob Agents Chemother 55:947-53.
